# Chromosome 12 and Environmental Factors in Parkinson’s Disease: An All of Us Data Analysis

**DOI:** 10.3390/genes16101197

**Published:** 2025-10-13

**Authors:** Kenta Abe, Karen Niemchick

**Affiliations:** School of Interdisciplinary Health, College of Health Professions, Grand Valley State University, 301 Michigan Street NE, Grand Rapids, MI 49503, USA; abek@mail.gvsu.edu

**Keywords:** Parkinson’s disease, chromosome 12, *PARK8*, *LRRK2*, GWAS

## Abstract

Background/Objectives: Parkinson’s disease (PD) is a neurodegenerative disease that develops with age and is related to a decline in motor function. Studies suggest that the causes may be based on genetic dysfunction including *PARK* gene mutations and environmental factors. Methods: To explore those factors, we used multivariable logistic regression to obtain odds ratios (ORs) and adjusted ORs by using the All of Us Dataset which contains genomic, blood test, and other environmental data. Results: On Chromosome 12, there were 3709 candidate genetic polymorphisms (GPs) that are associated with PD. Of those GPs, fourteen GPs had high ORs which are similar to the OR of the *PARK8* gene G2019S mutation. Of those 3709 GPs, a 2.00-fold change in OR was observed in five GPs located at bases 53,711,362 (OR = 4.86, 95% CI [1.46, 16.18]), 31,281,818 (OR = 4.37, 95% CI [1.02, 18.82]), 101,921,705 (OR = 5.38, 95% CI [1.23, 23.51]), 47,968,795 (OR = 7.82, 95% CI [1.81, 33.83]), and 112,791,809 (OR = 8.05, 95% CI [1.85, 35.05]) by calcium, Vitamin D, and alcohol intake and were statistically significant. Conclusions: The results suggest that the progression of some PD caused by certain GPs can be delayed or prevented by the environmental factors above. In February 2025, All of Us released the CT Dataset v.8 which has a 50% increase in the number of participants. Potentially, it may be possible to research more GPs and environmental factors. In future studies, we would like to explore other environmental factors and GPs on other chromosomes. It is believed that specific GPs may tailor current treatments and qualify patients for clinical trials. Additionally, genetic knowledge may help increase accuracy in clinical trials.

## 1. Introduction

Parkinson’s disease (PD) is a progressive neurodegenerative disorder [1]. PD is caused by a lack of dopamine due to the degeneration of the substantia nigra in the midbrain, which leads to impaired motor function, such as resting tremor, akinesia, and rigidity [2]. PD is more likely to develop with age [3], which also suggests that this may be related to a decline in brain function. In addition, PD affects more men than women in the United States. Females tend to be older than males at symptom onset [4]. While age and sex are major risk factors, the underlying cause of PD is unknown [1].

Why does brain dysfunction resulting in dopamine deficiency occur? To date, we know that there are genetic and environmental factors involved [2]. In the following section, we review previous research based on both genetic and environmental factors.

### 1.1. Epidemiology of Parkinson’s Disease

According to the World Health Organization, more than 8.5 million individuals had PD in 2019, doubling in the past 25 years [5]. In North America, the estimated prevalence range for those aged >65 is from 108 to 212 per 100,000 [6]. Furthermore, the economic burden is also a problem. In the United States, the estimated annual costs of PD is approximately USD 52 billion [7].

PD prevalence increases with age [8]. Because the worldwide population is aging, it is estimated that the number of individuals who have PD may grow to 12.9 million in 2040 [9]. The burden is also on caregivers. Between 2017 and 2019, average caregiver age was over 60, and 70% of caregivers were patients’ spouses [10]. This suggests that both PD patients’ and caregivers’ burden may be increasing due to worldwide aging. The problem of quality of life (QOL) is also crucial. Studies suggest that PD patients have significantly poorer QOL compared with healthy individuals, and this affects functional outcomes [11]. Regarding disability-adjusted life-years (DALYs) of PD, China, India, the United States, Japan, and Germany had the five highest prevalence rates and DALYs in 2019 [12]. Since DALY is calculated by the sum of years of healthy life lost due to disability and years of life lost due to premature mortality [13], the result means that the patients in the five countries above live with the burden for a long time.

### 1.2. Classification of Types of PD

PD is classified as monogenic PD and idiopathic PD, but care must be taken with this terminology, as the definition varies slightly depending on the researcher. The definition of monogenic PD is almost consistent, and is PD caused by mutations in single genes [1,14]. The term idiopathic, however, can have two main meanings depending on the context: one refers to the development of PD due to genome-wide association, i.e., multiple gene alterations [8,15], and the other refers to a combination of complex genetic and environmental factors [1,16]. Based on the first definition, 3–5% of PD can be explained as a monogenic cause and 16–36% can be explained by idiopathic [8], whereas based on the second definition, 10% of PD is a monogenic form and 90% is idiopathic [1,16]. In this study, we adopt the second definition because monogenic PD is a minority condition and, since its cause is unknown, factors other than genetic should also be considered.

### 1.3. Environmental Factors

Some have gone as far as to argue that most cases of PD are preventable by avoiding environmental exposure to chemicals such as certain pesticides, the solvent trichloroethylene, and air pollution [17].

#### 1.3.1. Diets

According to Maraki et al., there is a correlation between the onset of PD and certain foods [18]. The results of a cross-sectional study of 1731 elderly Greek people showed that those who ate a Mediterranean diet (MeDi) had a lower probability of developing prodromal PD. They also mentioned that the number of studies investigating the relationship between MeDi and PD is limited. However, in the context of diet and PD, the benefits of MeDi are often mentioned, many of which suggest a preventive effect [19,20,21].

One of the reasons why diet is related to PD is thought to be changes in intestinal bacteria. Regarding the progression of PD, there is the Braak hypothesis [22], which states that the pathological process of PD begins in the peripheral/enteric nervous system, progresses to the midbrain, and finally progresses to the cerebrum. According to a comprehensive narrative review by Omotosho et al., there is evidence suggesting a relationship between diet and PD, but they point out that not all PD can be explained by diet alone [23].

#### 1.3.2. Calcium

The association between PD and calcium intake is unclear. Previous studies show there is an association between PD and calcium [24], but some studies do not support the association [25]. More research is needed to confirm the association using epidemiological methods.

#### 1.3.3. Vitamin D

Vitamin D has a beneficial effect on calcium intake [26]. In the context of PD, studies show that PD patients have lower Vitamin D levels [27,28]. On the other hand, some conflicting results exist; one demonstrated an increased risk for PD with lower mid-life vitamin levels, and the other showed no association between Vitamin D levels and PD risk [29].

#### 1.3.4. Alcohol Intake

The relationship between alcohol consumption and PD is unclear. Alcohol, or ethanol, is contained in alcoholic beverages and consumed for recreational purposes [30]. Alcohol affects mainly the liver which metabolizes alcohol, but broad brain areas are also affected. Alcohol interacts with proteins involved in neurotransmission, but the interaction system is different from other drugs [31]. According to Kamal et al., some studies show that moderate alcohol consumption has a protective effect on PD, but others do not [32]. Therefore, it is necessary to confirm the association by using epidemiological methods.

### 1.4. Genetic Factors

Twenty-three *PARK* gene loci have been identified in the genome that are associated with the development of monogenic PD, but *PARK* gene mutations do not explain all PD cases [33]. The contribution of each *PARK* gene to hereditary PD varies from person to person. For example, *PARK1* is responsible for approximately 2% of hereditary PD cases [16]. *PARK2* mutation is responsible for approximately 50% [34] to as much as 77% [35,36] of early-onset PD, particularly in patients younger than 30 years of age. However, *LRRK2* mutations are the most common genetic cause of familial PD. The protein *α*-synuclein (*α*-syn), which is encoded by the *SNCA* gene, consists of 140 amino acids [37]. Although *α*-syn is thought to play an important physiological role, its detailed function remains unknown [38]. Monomeric *α*-syn is normally present throughout the brain [39]. However, when *α*-syn misfolds and becomes *α*-syn fibrils, it can cause damage to neurons and lead to cell loss [38]. Mutations in the *SNCA* gene can make *α*-syn more prone to misfold. Mutations such as the A53T mutation lead to autosomal-dominant PD [40]. The *SNCA* gene has been classified as *PARK1* (located at Chromosome 4), a genetic locus associated with PD.

*PARK2* encodes the Parkin protein, an E3 ubiquitin ligase [41]. Ubiquitin is a protein that can modify proteins, changing their function, or target them for degradation [42], and an E3 ligase is the enzyme that attaches ubiquitin [43]. Ubiquitin involves mitophagy, which is an autophagic process of damaged mitochondria. Thus, *PARK2* gene mutations lead to an accumulation of damaged mitochondria associated with the progression of PD [44].

In 1996, to identify the genetic locus associated with the development of PD, Polymeropoulos et al., examined genetic linkage analysis for a total of 140 genetic markers in a large Italian pedigree and identified the *SNCA* gene (*PARK1*) as being involved [45,46]. However, among the *PARK* genes identified using a genome-wide association study (GWAS), which is the gold standard for investigating genetic risk factors [47], some *PARK* genes cannot be determined clearly for the association with PD. For instance, Satake et al. identified a susceptibility locus thought to be correlated with PD in a GWAS of 2011 cases and 18,381 controls in Japan and reported it as the *PARK16* gene [48]. On the other hand, it has been reported that pathogenic mutations in the *PARK16* gene are rare in people of European descent [49], and some researchers have not yet added *PARK16* to the *PARK* group because the association has not yet been identified [33].

### 1.5. PARK8/LRRK2 Gene and Environmental Factors

There are multiple factors for PD, including monogenic genes, common variants, and environmental factors for PD. In this research, we will focus on *PARK8*/*LRRK2* gene single nucleotide polymorphisms (SNPs) and environmental factors.

The leucine rich repeat kinase 2 (*LRRK2*) gene is located on Chromosome 12. It was found in a large Japanese family as one of the causes of PD and identified as the *PARK8* gene in 2002 [50]. Studies suggest that if the 40,340,400th base of Chromosome 12 on the Genome Reference Consortium Human build 38 patch 14 (GRCh38.p14) [51] changes from G to A (G *>* A), also known as the G2019S mutation [52], a missense mutation results [53], leading to changes in the coded amino acid [54]. *LRRK2* missense mutations are the most common cause of monogenic PD but are relatively rare in the overall PD population. PD GWAS studies have identified genetic variants in and near the *LRRK2* gene as risk factors of PD in individuals who have no genetic cause of PD [53]. This suggests that it is important to investigate the relationship between GPs of not only the *LRRK2* gene but also other loci on Chromosome 12 and PD.

Moreover, although the relationship between PD and environmental factors has been mentioned in several previous studies, few studies have mentioned the interactions between the *LRRK2* gene mutation and Chromosome 12 GPs and environmental factors.

In this paper, unless otherwise noted, genomic base numbers follow GRCh38.p14.

### 1.6. Research Question

Due to the mix of genetic and environmental factors that confer risk for PD, the purpose of this research was to explore the relationship between genetic and environmental factors. Specifically, we are exploring (1) if the odds ratio of PD caused by the *PARK8* G2019S mutation is adjusted by calcium, Vitamin D, and alcohol intake, and (2) if there are other GPs found to be adjusted by calcium, Vitamin D, and alcohol intake.

## 2. Materials and Methods

The dataset uses genetic and environmental data provided by *All of Us*. The participants of *All of Us* are residents in the United States except those aged 5–18 (19 in Alabama, 21 in Puerto Rico), and individuals consented to sharing information about their privacy and genomics data [55]. The authors obtained permission to use the dataset from *All of Us* in September 2024.

This was a cross-sectional study using the *All of Us* Dataset [56]. The dataset contains short-read whole-genome sequencing (srWGS) data. By combining these with demographic and health-related data, we investigated the probabilities of having PD by genetic and environmental factors.

Probabilities of PD were estimated using adjusted odds ratios (AORs) using logistic regression. Since the terms of use forbid the output of *All of Us* raw data from the cloud server, the data was analyzed using Python 3.10.12 [57], Hail 0.2.130 [58], and Pandas 2.2 [59] on Jupyter Notebook 2.2.13 [60] running in the cloud server environment. More detailed methods are found at https://dx.doi.org/10.17504/protocols.io.dm6gpq7jdlzp/v1 (accessed on 17 September 2025).

### 2.1. Definition of GP in the All of Us Data

In the MatrixTable of Chromosome 12, data for all 1576,756 bases, 0/0 (reference type) was recoded as GP = 0, and all other combinations were recoded as GP = 1. There were 2429 types of GPs in the dataset. For all 245,394 participants, the total number of those with no GP was 381,055,807,656 bases (99.67%) and those with a GP was 1260,533,354 bases (0.33%). The total number of missing data was 17,086,615 bases.

### 2.2. Preliminary Analysis

The total sample size of the genomic data in the Controlled Tier Dataset was 245,394. In addition, the total sample size of *All of Us* Controlled Tier (CT) v.7 Curated Data Repository (CDR) was 245,388 (6 samples were dropped from the former genomic dataset [61]). In the latter sample, the total number of individuals with PD was 1422.

For preliminary analysis to find candidate GPs from 1576,756 bases, we used the former genomic data (*n* = 245,394), and 243,972 individuals (245,394–1422 = 243,972) were regarded as PD negative (case PD positive) = 1422 (0.58%), control (PD negative) = 243,972 (99.42%)). For the main analysis using specific candidate bases, we used the latter CDR which has 245,388 samples.

### 2.3. Power Analysis

#### 2.3.1. Simple Logistic Regression for PD and GPs

We used G*Power 3.1.9.7 [62] to analyze statistic power for a simple logistic regression. When the conditions are *α* = 0.05, 1–*β* = 0.80, the GP positive is 0.33%, PD positive is 0.58%, and the total sample size is 245,394; an OR of >2.57 can be detected statistically. Therefore, an OR of 2.58 was set as a criterion to extract candidate bases for preliminary analysis.

#### 2.3.2. Multivariable Logistic Regression for PD, GPs, and Environmental Factors

For an appropriate sample size of multivariable logistic regression, the number of events per variable (EPV) was proposed by Peduzzi et al. [63]. In the context of this study, it means how many in the sample are positive for PD. The number of variables refers to the number of independent variables. Peduzzi et al., pointed out that EPV should be 10 or more [63].

### 2.4. Data Processing

Table 1, Table 2 and Table 3 show the frequencies of all recoded variables. After the exclusion of missing values, the sample size was 34,162. The EPV for all dummy variables was 369/13 = 28.38, therefore the power analysis condition was fulfilled (note that 13 refers to the number of recoded variables except reference variables).

The age of each sample was calculated as a difference between birthday and 1 July 2022 which is the data cutoff date. Relatively, older people tend to have PD. Also, PD onset age less than 50 is regarded as young onset PD [64]. Additionally, in a previous study that analyzed genomic factors by using GWAS, the age of <50 years was used as the threshold of young-onset PD [65]. In the dataset of *All of Us*, the sample size of PD-positive under 50 years old was small. Therefore, it was recoded as binary (<50 and ≥50).

For sex, we chose “sex at birth” but not gender. The answers such as “Skip”, “No matching concept”, “I prefer not to answer”, “None”, and “Intersex” were recoded as missing. Then, male was recoded as 0 and female was as 1.

For calcium level, “calcium [mass/volume] in serum or plasma” was used. In the dataset, there were several unit types such as milligram per deciliter (mg/dL), millimole per liter, milligram per milliliter, no value, and others. Since some values of millimole per liter, milligram per milliliter, etc., seemed to be mistakes of mg/dL or other units and each sample size was relatively small, we used values that have mg/dL and others were regarded as missing data. Then, values of 10,000,000 mg/dL were deleted because they can be regarded as missing or mistaken. If one individual had multiple values since the individual took the blood test several times, the mean value was used. According to the University of California San Francisco, the normal value range of calcium levels is from 8.5 to 10.2 mg/dL [66]. Similarly, according to the National Institutes of Health, calcium level is typically 8.8 to 10.4 mg/dL in healthy people [67]. For the data of *All of Us*, quantile values were 9.00, 9.28, and 9.53 for Q1, Q2, and Q3. In the dataset, the sample size of >10 mg/dL seemed to be small; thus, from those data, we recoded the continuous values of the calcium level to 1 for <8.5, 2 for ≥8.5–<9.0, 3 for ≥9.0–<9.5, and 4 for ≥9.5.

For Vitamin D level, “25-hydroxyvitamin D3 [mass/volume] in serum or plasma” was used. In the dataset, there were several types of units such as nanogram per milliliter (ng/mL), milliliter per minute, pictogram per milliliter, and no value. We used values that have ng/mL and others were regarded as missing data. If one individual had multiple values since the individual took the blood test several times, the mean value was used. According to the University of Florida, 20–40 ng/mL or 30–50 ng/mL are recommended [68]. Similarly, according to Holic, <20 ng/mL is considered to be a Vitamin D deficiency, 21–29 ng/mL is considered to be insufficient, and 30 ng/mL to take full advantage [69]. For the *All of Us* Data, Q1 was 23.00, Q2 was 31.00, and Q3 was 39.33. Therefore, from the data above, we recoded Vitamin D values to 1 for <20 ng/mL, 2 for ≥20 ng/mL–<30 ng/mL, 3 for ≥30 ng/mL–<40 ng/mL, and 4 for ≥40 ng/mL.

For alcohol consumption level, “Alcohol: Drink frequency past year” was used. There were 5 levels; “Never”, “Monthly or less”, “2 to 4 per month”, “2 to 3 per week”, and “4 or more per week”. Each level was recoded as 0 to 4. Other answers such as “Prefer not to answer” and “Skip” were regarded as missing.

## 3. Results

### 3.1. PD and LRRK2 Gene GPs

We used the Hail logistic regression tool to calculate ORs for PD (positive or negative) and GPs (0 or 1) of all 1,576,756 bases. Results that had *p* > 0.050 and OR <2.58 were excluded. After the procedure, we obtained 3709 candidate bases.

The *LRRK2* (*PARK8*) gene is located between the 40,224,997th and 40,369,285th base of Chromosome 12 [70]. In the *LRRK2* gene, the 40,340,400th G > A SNP is associated with PD [52]. This mutation is called rs34637584 or G2019S on the Reference SNP (rs) Report.

In the 3709 candidate bases, 12 bases belonging to *LRRK2* were included. In the 12 bases, G2019S has the smallest *p*-value (OR = 5.46, 95% CI [2.90, 10.27], *p* = 0.000000139).

### 3.2. Other Bases That Have Similar ORs and p-Values of G2019S

Based on the result above, other bases that have similar ORs of PD and GPs were extracted (OR ≥ 5.00, 95% CI range ≤ 10). Table 4 shows the results that were used for comparison with the ORs of PD, GPs, and environmental factors, as described later.

### 3.3. PD and Environmental Factors

#### Factors and Statistical Power

In the *All of Us* data, there were 245,388 samples. This CDR included demographic data, health condition data, and questionnaire results.

Since some data were missing, too many multiple variables led to low EPV. The EPV should be over 10, therefore the sample size is too small to calculate ORs by using logistic regression. Moreover, in the last model which includes PD, GPs, and environmental factors, some environmental factors will be converted into dummy variables and some bases may include missing variables for GPs, therefore the sample size must be large. From this aspect, 5 variables (2 variables were from demographic data, 2 variables were blood test data, and 1 variable was from survey data) were chosen as environmental factors to keep the sample size more robust.

### 3.4. Adjusted ORs of PD, GPs, and Environmental Factors

We calculated (a) ORs of GPs for PD and (b) adjusted ORs (AORs) of GPs adjusted by environmental factors for 34,162 samples using the Hail logistic regression tool. Each sample had the data of GPs of 3709 candidate bases.

#### 3.4.1. Comparison of OR and AOR to Access the Environmental Factors’ Adjustment

From the result of 3709 bases, we chose AORs that had *p*-values < 0.050 and the difference between OR and AOR was ≥±2 units. Table 5 shows these results.

The AORs of GPs on the bases belonging to *CALCOCO1*, *SINHCAF*, and *DRAM1* were decreased and the AORs of GPs on the bases belonging to *TMEM106C* and *RPH3A* were increased.

*CALCOCO1* is a gene that works for DNA binding activity in specific RNA polymerase II cis-regulatory regions [71]. In the context of neurodegeneration, *CALCOCO1* dysfunction may contribute to Golgi homeostasis disruption that leads to neurodegenerative diseases, cancers, etc. [72]. This dysfunction can cause PD [73]. The *DRAM1* gene is associated with autophagy activation [74] and various tumors if the transcriptional expression is decreased [75]. The GPs of *DRAM1* may be associated with PD [76]. The *SINHCAF* gene adjusts cell migration [77]. One study found that *SINHCAF*’s dysfunction may contribute to Alzheimer’s disease [78], but few studies mentioned the relationship between *SINHCAF* and PD. The mutation of the *TMEM106B* gene on Chromosome 7, a homolog of *TMEM106C*, is a risk factor of neurodegeneration disease [79]. The *TMEM106B* gene can form amyloid filaments that cause neurodegeneration. Therefore, in this context, a *TMEM106C* mutation may be associated with PD. However, few studies mentioned the relationship between *TMEM106C* itself and PD. The protein encoded by the *RPH3A* gene may be involved in neurotransmitter release and the traffic of synaptic vesicles [80]. A study shows that loss of *RPH3A* functions contributes to dementia severity, cholinergic differentiations, and increased *β*-amyloid concentrations [81]. The *β*-amyloid oligomers may associate with *α*-syn oligomer formation, but typically *β*-amyloid itself has no association with PD [82]. Therefore, the association of the dysfunction of *RPH3A* and PD is unclear.

We also calculated the AORs of *LRRK2* (*PARK8*) G2019S and other GPs that had similar characteristics to G2019S. Table 6 shows the results. Results with *p*-value > 0.050 and uncalculatable were excluded. It is assumed that the cause of some ORs and AORs of GPs on certain bases could not be calculated because the sample size was changed from the preliminary analysis (*n* = 245,394) to the main analysis (*n* = 34,162) and each base has different missing samples.

For the *LRRK2* G2019S SNP, the difference between OR and AOR was 0.05. This result suggests that PD caused by the G2019S mutation receives few effects from age, sex, calcium level, Vitamin D level, or alcohol drinking habits. Also, for other GPs that had similar characteristics to G2019S, the differences between OR and AOR were relatively small and up to 1.60 units.

#### 3.4.2. AOR of GPs Adjusted by Environmental Factors

We calculated the AORs of each stratum of environmental factors based on the results referred to in Table 5. Table 7 shows these AORs. Each base has different missing data and those were excluded.

## 4. Discussion

### 4.1. Main Findings

In the preliminary analysis, we found 3709 candidate bases that may be associated with PD. In those bases, 14 bases had similar ORs of the G2019 SNP in *LRRK2* (*PARK8*) and some of the genes identified have not been previously associated with risk for PD [83,84,85,86,87,88,89,90,91]. Although those results were outside of the main focus of this study, they warrant additional investigation in future studies.

According to Shu et al., the ORs of PD caused by the G2019S mutation varied in each ethnicity, with an OR = 8.71, 95%CI [6.12, 12.38] (*p*-value < 0.000) among Europeans/West Asians [92]. Compared with the previous research, our ORs were lower. The Benjamini–Hochberg method was used to confirm the false discovery rate. As a result of FDR confirmation (total number of those tested: 3709), all 3709 results were significant.

In the results of the main analysis, there were two types of GPs: GPs which were not affected by adjustment for demographic and environmental factors, and those which were. The OR of the G2019 SNP on *LRRK2* was hardly adjusted by demographic and environmental factors, therefore individuals with G2019 should be regarded as people who may tend to have PD regardless of age, sex, calcium, Vitamin D, or alcohol intake. For PD related to other GPs that had similar ORs to the *LRRK2* SNP OR, the interpretation is similar.

However, ORs of PD caused by five GPs (chr12:53711362, chr12:31281818, chr12:101921705, chr12:47968795, chr12:112791809) were significantly changed by adjustment of demographic and environmental factors. Although the major factors of PD among the five GPs seem to be genetics, this result indicates that the susceptibility of PD caused by these five GPs may be modified by age, sex, and lifestyle related to calcium, Vitamin D, or alcohol intake.

Overall, for the five GPs above, AORs of age and sex were statistically significant. Therefore, it can be said that males over 50 years of age tend to have PD compared with younger males and women. This trend is consistent with previous studies.

The AORs of calcium level for the five GPs were less than 1 for high calcium levels and were statistically significant. However, it was not statistically significant for lower calcium levels. The result indicates that individuals with PD have lower calcium levels, but this result does not meet the results of previous studies [24,25]; thus more research is needed.

The AORs of Vitamin D levels for the five GPs were greater than 1 (95% CIs varied; from 1.02 to 2.64) for high Vitamin D levels and statistically significant. However, it was not statistically significant for lower Vitamin D levels. This result is opposite compared with previous studies indicating that PD patients have lower Vitamin D levels [27,28]. Objectively, Fullard and Duda concluded that there was no association between PD and Vitamin D levels [29]. Those three previous studies were systematic reviews, so the hierarchy of scientific evidence is higher than our cross-sectional study. However, since the results of previous studies were not consistent, more studies focused on Vitamin D levels are needed.

The results of alcohol consumption habits for the five GPs were statistically significant except for chr12:112791809 (*RPH3A*). For four GPs, the AORs indicated that alcohol drinking may reduce the odds of PD. However, Kamal et al., have shown that the association between alcohol and PD is unclear [32]; therefore, careful interpretation is needed. For the *RPH3A* SNP, alcohol consumption and PD were not associated statistically.

For the five GPs, *CALCOCO1* and *DRAM1* are associated with tissue metabolism and/or autophagy, which is crucial for reducing “garbage” substances such as amyloid beta and *α*-syn in the human brain. That dysfunction may be related to PD, but the results also show that the tendency can be reduced by environmental factors. Also, the result suggests that environmental factors may reduce the odds of PD related to the SNP on *SINHCAF* and increase the odds of PD related to the SNP on *TMEM106C*, but we could not find previous studies mentioning *SINHCAF* or *TMEM106C* for PD association; therefore, more study is needed.

### 4.2. Strengths and Limitations

There are some advantages found in this study. Firstly, this research studied the relationship between not only PD and GPs or PD and environmental factors but PD, GPs, and environmental factors; therefore, it suggests that some environmental factors and lifestyle can reduce the odds of PD even if the individual has certain GPs. Secondly, the results were statistically significant, and some results support previous studies. Thirdly, the differences between the OR and AOR of five GPs were compared to the results of the *LRRK2* gene as *PARK8*; therefore, the differences in characteristics between five GPs and *LRRK2* were shown.

Limitations in this study were that the results did not exclude the possibilities of confounders; our results of environmental factors mentioned only lifestyles related to calcium, Vitamin D, or alcohol intake. Also, this study did not mention the change in coding for each gene; not all GPs lead to the dysfunction of coded proteins, but the results did not take into account the above. Moreover, participants were divided into <50 and ≥50 years old, but since PD is associated with older age, more age groups in ≥50 may be needed.

Finally, the results of alcohol intake may have a strong bias because it is based on a survey and thus more likely to be inaccurate.

## 5. Conclusions

It is difficult to analyze live human brains for PD, but we can explore the correlation between PD, GPs, and environmental factors by using data. Some PD caused by gene mutations can be inevitable, but this study showed that some genetic PD risk is modifiable by nutrition intake or changing lifestyles.

It may also be said that PD is an ensemble-like neurodegeneration disorder played by many genomic factors. If some of the 3709 instruments named GPs are broken, the skewed harmony may lead to serious consequences. However, the results of this study suggest that if the conductor of the orchestra can intervene by using environmental factors, the consequences may be delayed or prevented. We used only calcium, Vitamin D, and alcohol intake levels for the proxy of environmental or lifestyle factors in this study, but the results can be used for further analysis of PD such as biomarkers to predict the progression of PD.

In the future, we would like to investigate the association of other factors with PD. We would also like to analyze interactions between GPs and GPs on other chromosomes so that we can specify more characteristics of PD, GPs, and environmental factors.

## Figures and Tables

**Table 1:** Recoded environment variables (*n* = 34,162).

Item	Category	Value	%
PD (n)	Positive	369	1.08
	Negative	33,793	98.92
Age (n)	<50	8310	24.33
	≥50	25,852	75.67
Sex (n)	Female	24,201	70.84
	Male	9961	29.16
Calcium (mg/dL)	<8.5	743	2.17
	≥8.5 and <9.0	4831	14.14
	≥9.0 and <9.5	17,183	50.3
	≥9.5	11,405	33.39
Vitamin D (ng/mL)	<20	5219	15.28
	≥20 and <30	9999	29.27
	≥30 and <40	10,394	30.43
	≥40	8550	25.03
Alcohol consumption (n)	Never	6771	19.82
	Monthly or less	11,244	32.91
	2 to 4 per month	7042	20.61
	2 to 3 per week	4714	13.80
	4 or more per week	4391	12.85

The sample size of 34,162 excludes missing data from all 245,388 samples.

**Table 2:** Cross table of Parkinson’s disease and recoded environment variables (*n* = 34,162).

Item	Category	PD Positive (*n* = 369)	PD Negative (*n* = 33,793)	*p*-Value
Age (n)	<50	9	8301	<0.000 ^1^
	≥50	360	25,492	
Sex (n)	Female	176	24,025	<0.000 ^1^
	Male	193	8301	
Calcium (mg/dL)	<8.5	8	735	<0.000 ^2^
	≥8.5 and <9.0	84	4747	
	≥9.0 and <9.5	180	17,003	
	≥9.5	97	111,308	
Vitamin D (ng/mL)	<20	28	5191	<0.000 ^2^
	≥20 and <30	85	9914	
	≥30 and <40	138	10,256	
	≥40	118	8432	
Alcohol consumption (n)	Never	103	6668	0.038 ^2^
	Monthly or less	115	11,129	
	2 to 4 per month	53	6989	
	2 to 3 per week	48	4666	
	4 or more per week	50	4341	

^1^ Pearson’s Chi-square test. ^2^ Mantel–Haenszel test for linear trend.

**Table 3:** Adjusted odds ratios of environmental factors for Parkinson’s disease (*n* = 34,162).

Item	Category	AOR (95% CI)	*p*-Value
Age (n)	<50	(Reference)	
	≥50	10.09 (5.18–19.64)	<0.000
Sex (n)	Female	0.41 (0.34–0.51)	<0.000
	Male	(Reference)	
Calcium (mg/dL)	<8.5	0.63 (0.30–1.32)	0.223
	≥8.5 and <9.0	(Reference)	
	≥9.0 and <9.5	0.61 (0.47–0.79)	<0.000
	≥9.5	0.49 (0.37–0.67)	<0.000
Vitamin D (ng/mL)	<20	0.70 (0.46–1.08)	0.112
	≥20 and <30	(Reference)	
	≥30 and <40	1.46 (1.11–1.92)	0.007
	≥ 40	1.56 (1.17–2.07)	0.002
Alcohol consumption (n)	Never	(Reference)	
	Monthly or less	0.89 (0.68–1.16)	0.389
	2 to 4 per month	0.59 (0.42–0.83)	0.002
	2 to 3 per week	0.73 (0.52–1.03)	0.077
	4 or more per week	0.66 (0.47–0.93)	0.016

AOR is the adjusted odds ratio. The sample size 34,162 is a result that excluded missing data from all 245,388 samples. The result was calculated by the StatsModels module on Python.

**Table 4:** Odds ratios of *LRRK2* G2019S and other genetic polymorphisms with similar odds ratios and 95% confidence interval ranges (odds ratio ≥ 5.00, 95% confidence interval ≤ 10 (*n* = 245,394, outcome: Parkinson’s disease positive/negative).

Base No. (GRCh38.p14)	OR (95% CI)	Original *p*-Value	Adjusted *p*-Value	Gene Name
1860203	5.58 (2.76–11.31)	<0.000	<0.000	*CACNA2D4*
4628152	5.43 (2.43–12.11)	<0.000	<0.000	*AKAP3*
11869533	5.94 (2.93–12.05)	<0.000	<0.000	*ETV6*
13537468	5.25 (2.97–9.27)	<0.000	<0.000	*GRIN2B*
30983164	5.53 (2.60–11.76)	<0.000	<0.000	*TSPAN11*
38321345	5.77 (2.85–11.69)	<0.000	<0.000	*ALG10B*
40340400 (G2019S)	5.46 (2.90–10.27)	<0.000	<0.000	*LRRK2 (PARK8)*
48569196	5.28 (2.61–10.70)	<0.000	<0.000	*LALBA*
49990203	5.09 (2.26–11.48)	<0.000	0.002	*RACGAP1*
52107506	5.33 (2.36–12.02)	<0.000	0.001	*SMIM41*
65955867	5.06 (2.24–11.42)	<0.000	0.002	*HMGA2*
66254622	5.87 (2.89–11.89)	<0.000	<0.000	*IRAK3*
81260048	5.30 (2.35–11.96)	<0.000	0.001	*ACSS3*
108544453	5.01 (2.48–10.15)	<0.000	<0.000	*SART3*
120460300	5.69 (2.67–12.10)	<0.000	<0.000	*GATC*

This is a part of the preliminary analysis resulting in 3709 bases. At the main analysis, those bases in Table 1 were compared with the main results using the *All of Us* CD (*n* = 245,388). Original *p*-values were calculated by logistic regression. Adjusted *p*-values are corrected *p*-values for the detection of false discovery rate (FDR) by using the Benjamini–Hochberg method. For FDR confirmation, StatsModels on Python was used.

**Table 5:** Five odds ratios of genetic polymorphisms for Parkinson’s disease adjusted by age, sex, calcium level, vitamin D level, and alcohol drinking habits factors (*n* = 34,162).

Base No. (GRCh38.p14)	OR (95% CI)	AOR (95% CI)	AOR *p*-Value	Gene Name
53711362	7.48 (2.30–24.37)	5.00 (1.51–16.59)	0.009	*CALCOCO1*
31281818	6.57 (1.56–27.69)	4.37 (1.02–18.78)	0.047	*SINHCAF*
101921705	7.67 (1.81–32.56)	5.51 (1.27–23.95)	0.023	*DRAM1*
47968795	4.97 (1.19–20.71)	7.83 (1.82–33.62)	0.006	*TMEM106C*
112791809	5.61 (1.34–23.44)	8.19 (1.89–35.46)	0.005	*RPH3A*

AOR is the adjusted odds ratio. Since the Hail logistic regression tool does not provide a dummy variable function and does not calculate the AORs of variables that are used for adjustment, we obtained only the AORs of GPs.

**Table 6:** Odds ratios of *LRRK2* (*PARK8*) and six genetic polymorphisms for Parkinson’s disease adjusted by age, sex, calcium level, vitamin D level, and alcohol drinking habits factors (*n* = 34,162).

Base No. (GRCh38.p14)	OR (95% CI)	AOR (95% CI)	AOR *p*-Value	Gene Name
1860203	4.77 (1.49–15.29)	4.60 (1.41–15.03)	0.012	*CACNA2D4*
13537468	5.68 (1.41–22.82)	4.52 (1.12–18.23)	0.034	*GRIN2B*
30983164	5.12 (1.59–16.45)	4.41 (1.35–14.45)	0.014	*TSPAN11*
40340400 (G2019S)	5.52 (2.00–15.21)	5.56 (1.99–15.57)	0.001	*LRRK2 (PARK8)*
49990203	5.89 (1.82–18.99)	4.29 (1.31–14.08)	0.016	*RACGAP1*
81260048	5.03 (1.57–16.45)	4.23 (1.30–13.83)	0.017	*ACSS3*
120460300	5.22 (1.62–16.77)	4.32 (1.33–14.05)	0.015	*GATC*

AOR refers to adjusted odds ratio. The results were calculated by Hail logistic regression tool.

**Table 7:** Adjusted odds ratios of the 53,711,362th base (*CALCOCO1*), 31,281,818th base (*SINHCAF*), 101,921,705th base (*DRAM1*), 47,968,795th base (*TMEM106C*), 112,791,809th base (*RPH3A*), and environmental factors.

Item	Category	chr12:53711362 (*n* = 34,162)		chr12: 31281818 (*n* = 34,158)		chr12: 101921705 (*n* = 34,150)		chr12: 47968795 (*n* = 34,144)		chr12: 112791809 (*n* = 16,972)	
		AOR (95% CI)	*p*-Value	AOR (95% CI)	*p*-Value	AOR (95% CI)	*p*-Value	AOR (95% CI)	*p*-Value	AOR (95% CI)	*p*-Value
GP	Reference nucleotide	(Reference)		(Reference)		(Reference)		(Reference)		(Reference)	
	Polymorphic nucleotide	4.86 (1.46–16.18)	0.010	4.37 (1.02–18.82)	0.048	5.38 (1.23–23.51)	0.025	7.82 (1.81–33.83)	0.006	8.05 (1.85–35.05)	0.005
Age (n)	<50	(Reference)		(Reference)		(Reference)		(Reference)		(Reference)	
	≥50	10.04 (5.16–19.55)	<0.000	10.08 (5.18–19.62)	<0.000	10.07 (5.17–19.60)	<0.000	10.10 (5.19–19.67)	<0.000	11.44 (4.22–31.01)	<0.000
Sex (n)	Female	0.42 (0.34–0.52)	<0.000	0.41 (0.34–0.51)	<0.000	0.42 (0.34–0.51)	<0.000	0.41 (0.33–0.51)	<0.000	0.55 (0.40–0.74)	<0.000
	Male	(Reference)		(Reference)		(Reference)		(Reference)		(Reference)	
Calcium (mg/dL)	<8.5	0.56 (0.26–1.21)	0.014	0.64 (0.30–1.32)	0.227	0.64 (0.31–1.33)	0.227	0.64 (0.30–1.32)	0.226	0.66 (0.20–2.16)	0.489
	≥8.5 and <9.0	(Reference)		(Reference)		(Reference)		(Reference)		(Reference)	
	≥9.0 and <9.5	0.61 (0.47–0.79)	<0.000	0.61 (0.47–0.79)	<0.000	0.61 (0.47–0.79)	<0.000	0.61 (0.47–0.79)	<0.000	0.68 (0.46–1.00)	0.050
	≥9.5	0.49 (0.37–0.67)	<0.000	0.50 (0.37–0.67)	<0.000	0.49 (0.37–0.67)	<0.000	0.49 (0.36–0.66)	<0.000	0.50 (0.32–0.78)	0.002
Vitamin D (ng/mL)	<20	0.71 (0.46–1.09)	0.118	0.71 (0.46–1.09)	0.115	0.71 (0.46–1.09)	0.114	0.72 (0.46–1.10)	0.130	0.50 (0.24–1.03)	0.059
	≥20 and <30	(Reference)		(Reference)		(Reference)		(Reference)		(Reference)	
	≥30 and <40	1.44 (1.10–1.90)	0.009	1.45 (1.11–1.91)	0.007	1.45 (1.11–1.91)	0.007	1.48 (1.13–1.95)	0.005	1.52 (1.02–2.26)	0.040
	≥40	1.55 (1.16–2.05)	0.003	1.55 (1.17–2.06)	0.002	1.56 (1.17–2.07)	0.002	1.59 (1.20–2.11)	0.001	1.76 (1.18–2.64)	0.006
Alcohol consumption (n)	Never	(Reference)		(Reference)		(Reference)		(Reference)		(Reference)	
	Monthly or less	0.89 (0.68–1.17)	0.397	0.89 (0.67–1.16)	0.377	0.89 (0.68–1.17)	0.394	0.89 (0.68–1.16)	0.391	0.99 (0.67–1.46)	0.961
	2 to 4 per month	0.59 (0.42–0.83)	0.002	0.59 (0.42–0.83)	0.002	0.59 (0.42–0.83)	0.002	0.58 (0.41–0.81)	0.002	0.66 (0.41–1.06)	0.085
	2 to 3 per week	0.71 (0.50–1.01)	0.060	0.73 (0.52–1.04)	0.079	0.73 (0.51–1.03)	0.071	0.73 (0.51–1.03)	0.072	0.66 (0.39–1.13)	0.128
	4 or more per week	0.65 (0.46–0.92)	0.015	0.66 (0.47–0.93)	0.016	0.66 (0.47–0.93)	0.016	0.66 (0.47–0.93)	0.017	0.70 (0.43–1.16)	0.165

AOR refers to adjusted odds ratio. For the 53,711,362th base, missing samples were 16, EPV = 28.31 ≥ 10; for the 31,281,818th base, missing samples were 4, EPV = 28.38 ≥ 10; for the 101,921,705th base, missing samples were 12, EPV = 28.38 ≥ 10; for the 47,968,795th base, missing samples were 18, EPV = 28.31 ≥ 10; for the 112,791,809th base, missing samples were 17,190, EPV = 13.77 ≥ 10.

## Data Availability

More detailed methods are found at https://dx.doi.org/10.17504/protocols.io.dm6gpq7jdlzp/v1 (accessed on 17 September 2025).

## Abbreviations

The following abbreviations are used in this manuscript:

PD	Parkinson’s disease
OR	Odds ratio
AOR	Adjusted odds ratio
AD	Alzheimer’s disease
GRCh38.p14	Genome Reference Consortium Human build 38 patch 14
